# Saccades predict and synchronize to visual rhythms irrespective of musical beats

**DOI:** 10.1080/13506285.2018.1544181

**Published:** 2018-12-04

**Authors:** Jonathan P. Batten, Tim J. Smith

**Affiliations:** Department of Psychological Sciences, Birkbeck, University of London, London, UK

**Keywords:** Saccadic timing, audiovisual, music, entrainment

## Abstract

Music has been shown to entrain movement. One of the body’s most frequent movements, saccades, are arguably subject to a timer that may also be susceptible to musical entrainment. We developed a continuous and highly-controlled visual search task and varied the timing of the search target presentation, it was either gaze-contingent, tap-contingent, or visually-timed. We found: (1) explicit control of saccadic timing is limited to gross duration variations and imprecisely synchronized; (2) saccadic timing does not implicitly entrain to musical beats, even when closely aligned in phase; (3) eye movements predict visual onsets produced by motor-movements (finger-taps) and externally-timed sequences, beginning fixation prior to visual onset; (4) eye movement timing can be rhythmic, synchronizing to both motor-produced and externally timed visual sequences; each unaffected by musical beats. These results provide evidence that saccadic timing is sensitive to the temporal demands of visual tasks and impervious to influence from musical beats.

People move to music, from the involuntary foot taps of headphone wearing commuters, to full body movements on a dance floor. These commonplace occurrences demonstrate a capacity of music to entrain movement in humans, both spontaneous movements like tapping and head-nodding, to full body actions (Burger, Thompson, Luck, Saarikallio, & Toiviainen, 2014; Large, 2000). When we move resonates with a perceived beat, an underlying pulse extracted from the accents of the music (Patel & Iversen, 2014). Perceiving musical beats engages a wider network than audition alone, it is integrally linked with neural activation of motor areas (Stupacher, Hove, Novembre, Schütz-Bosbach, & Keller, 2013). Changes in the frequency of physiological movements including heart-rate and respiration can be induced by varying the tempo of background music (Khalfa, Roy, Rainville, Dalla Bella, & Peretz, 2008), in addition to the pace of motor actions, for example the speed of walking (Moens et al., 2014). Considering these multimodal entrainment effects of rhythmic body movement to musical beats (and the broader notion that perception is multimodal), in this present study we endeavoured to investigate the impact of music on one of the human body’s most frequent motor actions, saccadic eye movements, which are a viable candidate due to their highly systematic timing.

Throughout our waking lives, human gaze reorients spatially with a ballistic movement (i.e., saccade) around three times per second (Henderson, 2003). Eye movement research has predominately focused on the relationship between the spatial composition of visual features in a scene, and how they influence where gaze is directed in that scene (Itti & Koch, 2000; Itti, Koch, & Niebur, 1998; also see Borji & Itti, 2013, for a recent review). Recent interest has turned to the timing of gaze shifts (Einhäuser & Nuthmann, 2016; Henderson, 2007; Nuthmann, 2016; Tatler, Brockmole, & Carpenter, 2017). The length of time gaze remains stable at a particular spatial location (i.e., fixation duration) varies, and the timing of eye movements is subject to both direct control as well an automated (potentially rhythmic) routine (Findlay & Walker, 1999). Both cognitive and visual features can alter the duration of fixations, for example visually degraded images increase fixation durations (Wooding, Ruddock, & Mannan, 1995), and conducting a visual search generates shorter fixations than a memorization task of the same scene (Henderson, Weeks, & Hollingworth, 1999; Luke, Smith, Schmidt, & Henderson, 2014). Recent theories of eye-movement timing argue for an automatic stochastic timer, which generates eye movements independent of currently fixated visual information or sequential spatial targeting (Findlay & Walker, 1999), although competing models without a timer exist, (see Reichle, Pollatsek, & Rayner, 2012). In addition to the timer, there is a visually sensitive dynamic process-monitoring inhibitory mechanism, which can delay or cancel saccades (Engbert, Nuthmann, Richter, & Kliegl, 2005; Nuthmann, Smith, Engbert, & Henderson, 2010; Yang & McConkie, 2001) if they occur during an early stage of programming, known as the *labile* stage (Becker & Jürgens, 1979). These inhibitory (or cancellation) decisions are made based on the processing demands of the currently fixated visual information (Henderson & Pierce, 2008; Henderson & Smith, 2009; Yang & McConkie, 2001), although these behaviours have been characterized using static images. The two main influences on eye movement control are observed when employing the stimulus onset delay paradigm (SOD), which manipulates the delay in presentation of a static visual scene at key fixations in a systematic way, for an example see (Henderson & Smith, 2009). The SOD generates a bimodal distribution of fixation durations, observed as fixations that are sensitive to the visual presentation delay and increase linearly with increasing delay (the inhibition mechanism), and a visually insensitive quicker series of fixations indicative of a stochastic automatic timer (see Nuthmann et al., 2010 for a comprehensive review and computational model). A further feature of eye movements highlighted in the SOD paradigm and complemented by the body of work on micro-saccades (Martinez-Conde, Otero-Millan, & Macknik, 2013) is that the eyes never stop moving; the timer will eventually execute a saccade. A logical conclusion that could be drawn from these studies is that in a controlled visual environment, which limited the visual and task variance known to slow or cancel eye movements (factors that promote direct control), the produced sequence of eye movements would be minimally variant, rhythmic, and indicative of the timer. What is unclear is (1) whether other internal timing mechanisms (e.g., beat perception) dynamically influence the pacing of this rhythmic movement, and (2) how sensitive this timing is to dynamic visual onsets.

Musical beats are salient and predictable moments in time that commonly resonate with a natural pace of movement (McKinney & Moelants, 2006). The close relationship between musical beat perception and the timing of motor actions has been shown behaviourally in multiple studies that explicitly task people to synchronize a movement, commonly a finger tap with a beat (see Large, 2000; or Repp & Su, 2013, for a detailed review). Whilst there are individual differences in finger-tap timing ability, most notably between those with musical training and those without (Kraus, 2011; Tierney & Kraus, 2013), a majority of participants are able to achieve a relatively high level of synchronization. The close link between movement and auditory beat perception not only facilitates the ability to generate accurate temporal movement, but also suggests a highly effective predictive process that is able to programme and align motor movements to coincide with a future externally generated beat (Patel & Iversen, 2014). Much of the research regarding beat perception has focused on the ability to explicitly beat match a movement with tones in a sequence, less emphasis has been directed to the spontaneous production of synchronous movements, otherwise known as *implicit entrainment*. There is some evidence of background music implicitly influencing the timing of rhythmic behaviour beyond the physiological and walking research previously mentioned (Khalfa et al., 2008; Moens et al., 2014). For example Kuribayashi and Nittono (2015) found that when completing a simple line drawing task, variance in the tempo of background music linearly altered the pace of line drawing (although the timing of these movements was not synchronized with the beat). Somewhat contradictorily, there is also evidence that intention to synchronize is essential to reliably produce synchronized movements with music (Leow, Waclawik, & Grahn, 2018; Repp & Su, 2013). Taken together with the explicit beat matching tasks, there is clear evidence of a relationship between the perception of a musical beat and the timed execution of rhythmic movement, although evidence that musical beats implicitly affect movement is mixed.

The utility of predictable moments in time for efficient action is supported by the dynamic attending theory (Jones & Boltz, 1989), which argues that regular temporal cues are used to predict future events. This predictive ability facilitates or entrains the dynamic modulation of cognitive resource to enhance the predicted salient moments in time. This modulation allocates “energy” or the onset of attention in time. As the locus of visual attention is highly correlated with where gaze is allocated (Deubel & Schneider, 1996; Kowler, Anderson, Dosher, & Blaser, 1995), and eye movements are subject to some voluntary control, the entrainment of visual attention to a musical beat should coincide with saccadic movements that serve to optimize the perceptual processing of visual information, ultimately regulating the timing of eye movements to synchronize with a predictable beat. Evidence in favour of the dynamic attending theory has been observed in studies that have found an on-beat perceptual advantage when visual events are aligned with a predictable external auditory beat. A study by Van der Burg, Olivers, Bronkhorst, and Theeuwes (2008) demonstrated an increase in the perceived visual salience of a target within a search array when the presentation synchronized with an auditory tone (a ‘pip’). The enhanced visual salience (a “pop out”) was evident as quicker detection of an ambiguous target. A second study by Escoffier, Sheng, and Schirmer (2010) tasked participants with a visual discrimination task, which involved quickly identifying whether an image of a face or house was upright or inverted. The task was conducted with background music or in silence, and when the images were presented with music the timing was either in or out of synchrony with the beat. The authors observed that reaction times to images presented with the musical beat were faster than those presented off-beat or in silence. They argue that visual attention was entrained to the beat of the music, creating salient moments in time*.* The dynamic influence of entrained visual attention on eye movements would be most apparent when gaze shifts are required to access a dynamic presentation of sequential visual information. Therefore, a *gaze-contingent* sequential presentation could test whether eye movement timing is actively modulated by the entrained dynamics of visual attention (which is sensitive to musical beat), and if this is employed to enhance visual perception at predictable moments in time.

Within natural pacing limitations, when the eyes move is subject to some direct control. Hornof and Vessey (2011) found that the ability to synchronize horizontal saccades required an inter-onset-interval (IOI) range from 250 to 500 ms (240–120 BPM), which is notably restricted when contrasted to finger tapping with IOIs ranging from 200 to 2400 ms (300–25 BPM; see Repp & Su, 2013 for a review). Importantly, their task involved no visual processing demands. The evidence of implicit musical influence on eye movement timing is more limited. A study by Schäfer and Fachner (2014), eye-tracked participants who free-viewed either a static scene of a house, or a first person film of a car driving down an empty road. The visuals were presented with background music or in silence. In both scenes (static and dynamic), the authors found a significant increase in participant’s fixation durations when viewing with music, which may suggest an implicit musical influence on the timing of eye movements (although this is unlikely to be entrainment as the timing changes were not matched to the actual beat changes). A further eye-tracking study by Day, Lin, Huang, and Chuang (2009), directly measured eye movements subject to variation in musical tempo when completing a decision making task. The authors observed differences in decision-making accuracy subject to increasing tempo (a faster tempo increased accuracy and response time), although the finding of interest to this study was in the exploratory analysis. They found a significant increase in the frequency of eye movements when listening to fast rather than slow music. There is, therefore some evidence of musical influence on eye movements, both when explicitly controlled and implicitly affected. Although to fully identify music as an entraining force requires systematic control of visual influences on eye movement timing that these post-hoc analyses did not have.

To facilitate a controlled investigation into eye movement timing requires design considerations that maximizes the number of similar saccades produced whilst minimizing known sources of variance. Both the content and complexity of visual information can alter eye movement timing (Luke et al., 2014; Wooding et al., 1995) as well as saccadic programming decisions, for example, direction or amplitude changes (Honda & Findlay, 1992; Ludwig & Gilchrist, 2003; Smith & Henderson, 2009). These effects were minimized in the studies presented here by generating a novel visual task that induces a pattern of eye movement that is equal in amplitude from fixation to fixation, constant in location, and predictable in sequence with minimal reversals in gaze direction (e.g., due to reaching the screen edge). The external timing demands in the visual information were also simplified by employing either a gaze-contingent window, which dynamically triggered visual changes based on the eye movement itself (e.g., Nuthmann & Malcolm, 2016), a self-paced visual transition controlled by finger taps on a keyboard, or an externally timed visual sequence.

The intention for these studies are threefold, first, to investigate the entraining influence of musical beats on eye movements across three different visually rhythmic conditions: gaze-contingent timing (i.e., no peripheral visual transients; musical rhythm only) and motor-contingent timing (experiment 1), and when visual transitions are externally timed (experiment 2). Secondly, to measure the capacity for intentional synchronization, i.e., when the timing of each eye movement is volitionally controlled to match musical beats (experiment 1). Finally, to investigate the rhythmic nature of the saccadic timer as a phased response to controlled visual and audiovisual sequences using circular statistics. In other words, are eye movements predictive, reactive or independent of visual timing sequences and does the addition of musical beats influence this?

## Experiment 1

This first study will contrast eye movements across two *orienting modalities*, gaze-contingent and finger-tap contingent visual transitions, both with musical beats (three tempi / *musical IOI*) and in silence, and across two different *tasks,* either explicit*,* synchronizing each movement to the music, or completing the task with irrelevant background music (implicit). An initial prediction is that the intention to synchronize will be evident in eye movement timing in the explicit task as within their natural pacing bounds both eye movements and finger taps can explicitly synchronize some movement with an auditory beat (Hornof & Vessey, 2011; Repp & Su, 2013). The second prediction is that the on-beat dynamic allocation of attention (Jones & Boltz, 1989), as observed in Escoffier et al. (2010), will coincide with execution of eye movements, aligning them to synchronize with the musical beat when implicitly listening to music, and as in Day et al. (2009), when the interval between the beats in the music shortens the latency of eye movements will decrease. A final prediction is that the synchronization of eye movements in the tap-contingent condition will be closer aligned in phase with the music than the gaze-contingent movements. The tap-contingent condition has both a motor and visual transient information and the gaze contingent condition depends solely on self-awareness of eye movements, which is known to be limited (Clarke, Mahon, Irvine, & Hunt, 2016).

## Method

### Participants

A power analysis using G*Power (Faul, Erdfelder, Lang, & Buchner, 2007) of our prior related study, using the same paradigm (Batten, Dick, & Smith, 2016) which had a main effect of explicit synchronization (*η^2^* = .627) on eye movement timing, was performed. This effect size was used to inform an appropriate sample size (*N* = 10) for this study powered to *d *= .95 when *α* = .05. As an intention was to measure implicit entrainment, which is likely to have a smaller effect, the target sample size was increased to 30. The study tested 39 participants. Of these, 9 were unable to either consistently tap a finger in time (5/9, when finger-tapping was the only task) or had incomplete eye tracking data due to calibration difficulties following a mid-experiment break that meant continuing with the experiment was not possible (4/9). This left 30 participants (22 Female), with an age range from 18 to 32 (*M* = 22.5, SD = 4.26), who contributed data for analysis. Musical training scores generated from a factor in the Goldsmith Musical Sophistication Index (Müllensiefen, Gingras, Musil, & Stewart, 2014), with a scale from 7: no training to 49: more training than 95% of the population, and indicated a broad range of training from 11 to 43 (*M* = 24.8, SD = 9.3); none of the participants were professional musicians. There was a positive (although non-significant) relationship between musical training score and synchronization of finger-tapping performance (*p *= .18). Those with more training had a slightly more synchronized response (this measure is described within 1.2.5), although lack of a significant correlation is likely limited by the participation criteria which required (and positively recruited) those with a good tapping or beat production ability.

### Apparatus

Monocular eye movements of the participant’s dominant eye were captured using a desktop-mounted EyeLink 1000, recording at 1000 Hz with participant’s head mounted on a chinrest. The visual stimuli were presented on an ASUS VG248QE 24-inch screen, with the resolution set to 1024 × 768 pixels at 144 Hz (with black vertical boxing limiting the display area). The subtended visual angles of the display area were 39.17° horizontal and 29.95° vertical. The experiment was built and displayed using Experiment Builder (SR Research) and analysed using the EyeLink Data Viewer defaults for fixation, blink and saccade classification (e.g., a saccade had a minimum velocity of 30° / second). The musical beats were played in mono through Beyerdynamic DT-770 PRO 80 OHMS over-ear headphones pre-set to a comfortable volume.

### Stimuli

**Figure 1.**
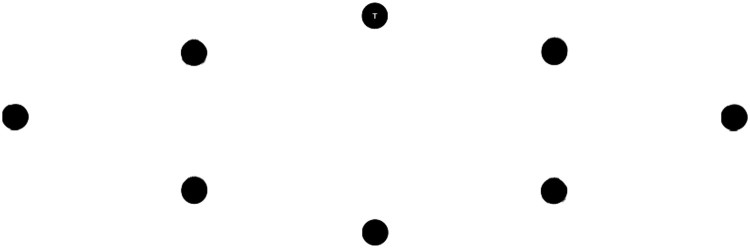
The elliptical visual stimuli array. The black reference circles were always visible. A single letter (either T or L) was displayed within the centre of a circle (as in the top circle) at all times.

The aim of the visual search paradigm was to produce a sequence of movements that were equal in size, distance, visual processing demand and minimally variant in saccade direction. Earlier versions of this visual search task utilized a circular design (Batten et al., 2016), although analysis of the saccade durations when orienting around the circular shape found that vertical saccades, especially downward saccades were significantly slower than those that are horizontal. This effect of saccade direction has been identified before (Honda & Findlay, 1992; Ludwig & Gilchrist, 2003). Therefore we employed a maximally horizontal elliptical shape which did not have this directional^1^ effect (the shape is shown in Figure 1). In controlling for these factors (size, distance, angle, visual complexity, and the timing of visual information) the elliptical search paradigm was designed to produce an increasingly narrow distribution (minimal deviation) of quick and rhythmic eye movements, that could be matched by (and entrained to) auditory beats.

Each circle in the elliptical array, shown in Figure 1, was 1.2° in diameter. The distance between each circle was 7.8°. The longest letter edge of the T and L letter within each circle was 0.3°. These letters were chosen for their similar visual features and because distinguishing between them required observing detail (i.e., foveal vision). The presentation of a letter within a circle in the gaze-contingent condition was dependent on eye movement behaviour. A letter (T or L) was displayed within each circle sequentially as the gaze shifted sequentially clockwise around each circle in the array (starting at the top circle). Eye movements to a circle out of sequence (to non-circle regions or to the incorrect circle, e.g., not sequentially clockwise) did not change the display. That is, the prior circle continued to display the current letter until the correct circle was oriented to. An unseen interest area (IA) with a 3° diameter around each circle was used to classify eye movements as within each location. The presentation of a letter occurred after the participant’s gaze was contained within the bounds of the IA for 15 ms. This condition produced an imperceptible delay in presentation which ensured a fixation within each region. The tap-contingent condition required a key press to present the next letter, either “V” when L was displayed or “B” when T. The keys (B and V) are adjacent on a keyboard and the association was quickly formed in a practise trial (i.e., L is the left “V” key). Incorrect key presses (e.g., pressing the B key when L was displayed) did not change the current display, but were recorded as miss-hits for end-of-trial feedback on performance to ensure task compliance.

The musical stimuli were three intervals of a simple drum rhythm which corresponded with the natural pace of eye movements when completing the task in silence. The music was formed of alternating electronic style kick-drum and a snare-drum sound at equal intervals with matched intensity (e.g., kick, snare, kick snare). Each sound acting was an accent of the inter-onset interval (IOI) or beat. The simple music was percussive with no melody or variant metre and had an aesthetic similar to minimalist electronic music or an arcade game soundtrack. The decision to use this simple musical rhythm as opposed to metronomic stimuli was to make the experiment more enjoyable and game like. The three inter-onset intervals (tempi) of the music were informed by a pilot study on five naïve participants orienting around the elliptical shape in silence (1 practice trial and 5 trials for analysis), identifying infrequent changes in letter (between 28 and 31 circle movements before a change, from 5 to 7 changes per trial). The latency of eye movements (each fixation duration combined with the duration of the previous saccade) whilst completing the task was used to inform the IOI based on the revised visual demands. The mean latency was 293.32 ms, with a standard deviation of 29.51 ms. Therefore, the three musical IOI levels based on the 8-circle elliptical design were 264, 293 and 323 ms (227, 204 and 186 beats-per minute).

### Procedure

Participants were informed that the search task was identifying infrequent changes in letter sequences. Following informed consent each participant completed a short-version of the Edinburgh Handedness inventory (Veale, 2013), to identify which hand was dominant for the key-press responses. All keyboard and tap responses were made with the index finger on their dominant hand. The orienting mode around the ellipse was either gaze-contingent, looking at the next circle (clockwise) displayed the next letter; or tap-contingent, pressing the correct key for the currently displayed letter changed the letter presentation to the next circle clockwise (*V* for **L**, and *B* for **T**). When the mode was gaze-contingent, the task was to press the identifying letter on the keyboard (the key that corresponded to the changed letter, e.g., pressing V if the new letter sequence was L) as soon as a letter change occurred (the letter changes were infrequent occurring after 28–31 circle movements). When tap-contingent, the task required a correct key-response (using the index finger on their dominant hand) to progress. To encourage accurate response the number of incorrect key presses were shown at the end of each trial. The trial ended when a letter had been displayed around 180 circles or timed out after three minutes.

Both modality types began with the implicit task, completing the visual search task with irrelevant background music at the three IOI levels. After the implicit trials, the participants were given further instructions for the explicit task: to control their movements from circle to circle to match the beats in the music (every beat in sequence). The condition order of the orienting mode (gaze or tap) was counter-balanced across participants. Those in condition A completed each of the four trials (each IOI and a silent randomized trial) in a set order: implicit gaze, implicit tap, explicit gaze, and ending with explicit tap. Those in condition B began with tap trials and ended with gaze. For each condition there was a practice trial to ensure compliance with the instructions. There was also a randomly inserted silent trial in both the implicit and explicit blocks for each modality, during which the participants simply completed the visual search task. Therefore, each participant completed twenty trials, one practice trial followed by four trials for each condition. The experiment ended with two final tasks, first, taping in time to each IOI level 60 times per trial on an Apple track-pad. Secondly, to complete the musical training questionnaire (Müllensiefen et al., 2014). The total duration of the experiment was 50 min.

### Data analysis plan

Some data cleaning steps were taken with the eye movement data. Firstly, fixation classifications that were preceded by, prior to, or contained a blink were removed. Secondly, the fixations that did not follow the clockwise sequence (off target) were removed. For an eye movement to be included in the analysis the previous saccade was required to begin one circle prior and end one circle clockwise around the ellipse. This removed all re-fixations within the current interest area. Finally, very short fixation durations (less than 100 ms) and those greater than 1500 ms (indicative of off-task behaviour) were removed. These cleaning steps removed 31% of fixations, leaving an average of 162 fixations per level of each condition for each participant for analysis. Following these cleaning steps, each fixation was summed with the preceding saccade duration to generate the eye movement latency measure which is representative of the time interval of movement between the circles.

A goal of this study was to identify a measure of entrained response in eye movement timing to the three musical IOI. The relationship of interest is the timed responses as they align with the period and the phase of the beat interval (IOI). Therefore the eye-movements were analysed using both measures of period (latency) and phase, using circular methods, specifically the mean resultant length (MRL) and circular mean (Fisher, 1995) with the “circular” package for R (Agostinelli & Lund, 2017). Circular methods are particularly informative when synchronization performance is poor (Repp & Su, 2013).

As the null hypothesis is also plausible for predictions of musical influence, this paper will complement the use of frequentist statistics with Bayesian evidence for the null hypothesis where appropriate (a likelihood ratio called the Bayes factor, expressed as *BF_01_*). A Bayes Factor greater than 3 is generally considered strong evidence (Kass & Raftery, 1995).

**Figure 2.**
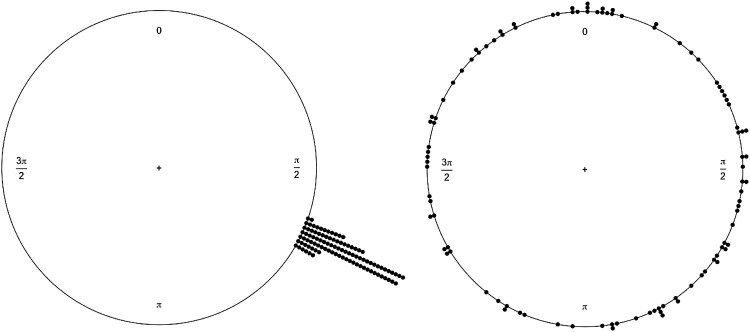
Two circular plots of simulated data (100 responses) as an example of synchronized responses with an MRL of .99 (left), where dots are individual responses to an isochronous rhythm, and the right are random or uniform responses with a very low MRL of .09. Rayleigh’s test is significant for the left but not the right.

The MRL is a deviation measure of the circular mean and is indicative of how tightly clustered response vectors are to the circular mean based on a pre-set phase (the musical IOI). The MRL has a range from 1 (all the vectors are equal to the circular mean), to 0 (a random distribution whereby the vectors bear no relationship to the circular mean), visualized in Figure 2. A statistical test of the MRL values, the Rayleigh test (Wilkie, 1983), confirms if the clustering of the vectors (MRL) within a condition are significantly representative of the circular mean (i.e., significantly synchronized). The eye movement representation that was coded for the timed response was the fixation start time (the end of a saccade). This timepoint was the most accurate representation in Hornof and Vessey (2011), and has been employed in other saccadic sensorimotor-synchronization tasks (e.g., Joiner & Shelhamer, 2006; Shelhamer & Joiner, 2003). This timepoint of an eye movement best parallels an entrained motor movement, i.e., when the finger lands is the timepoint aligned with the beat. An MRL value was generated and tested for significance (Rayleigh test) for each participant at each level of each condition. In conditions where most MRL values were significant further analysis contrasted the circular mean, that is where in phase the responses averaged.

## Results

### Latency

The musical IOI used in this experiment was generated from the latency distributions of five subjects who completed a silent pilot study. To confirm that the employed musical IOI matched the natural pace of the participants’ eye movements during this experiment, we employed a Bayesian one-sampled t-test to compare the gaze latencies from the silent trials (*M* = 291.1 ms, SD = 27.8) to the pilot mean latency (293.32 ms). There was positive evidence for the null, *BF_01_ *= 4.70 (*t*(29) = .444, *p* = .66), and confirmation that the musical IOI employed represented the mean latency of eye movements for this sample.

**Figure 3.**
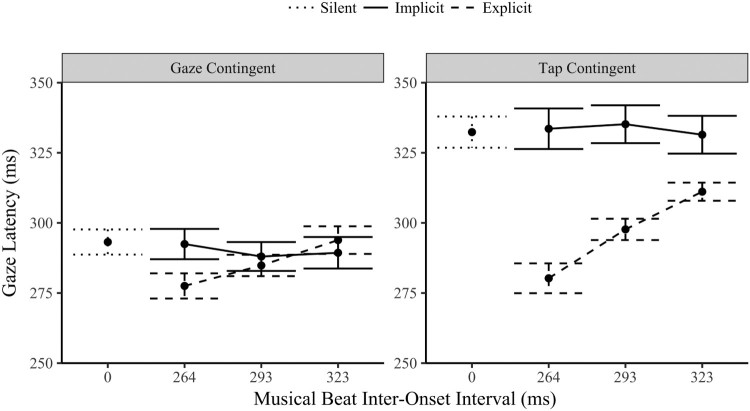
Gaze latencies (ms) ±1 SE by orienting modality (tap or gaze contingent eye movements), task (implicit or explicitly entrained movement) and musical IOI (264, 293 and 323 ms).

An initial test of whether the music implicitly influenced the timing eye movements in both the tap and gaze contingent conditions was to contrast the gaze latency of the three music IOI levels to the silent trials. As is apparent in Figure 3, Bonferroni corrected t-tests found no significant difference between the silent mean gaze latencies and those in the implicit condition (all *p* > .3). This evidence for invariance was further confirmed with Bayesian statistics. In the tap-contingent condition the gaze latencies had positive evidence for the null hypothesis (all *BF_01_* > 4.4). The Bayes factors for the three gaze-contingent IOI levels were less strong, but again provided evidence of invariance from the silent condition (264 IOI: *BF_01_* = 5.0; 293: *BF_01_* = 1.2; 323: *BF_01_* = 2.7). The presence of music did not significantly alter the latency of eye movements from silent for either orienting modality or musical IOI level in the implicit condition.

A three-way within-subject ANOVA compared the gaze latencies (fixation duration combined with the previous saccade duration) across the two modalities (gaze-contingent and tap-contingent), to the two tasks (implicit and explicit synchronization) and the three musical IOI levels (264, 293 and 323 ms). There was a significant main effect of orienting modality, *F*(1,29) = 59.1, *p* < .001, *η*^2^ = .671; Task, *F*(1,29) = 23.5, *p* < .001, *η*^2^ = .447; and musical IOI, *F*(2,58) = 12.2, *p* < .001, *η*^2^ = .296. The three-way interaction between modality, task and musical IOI was not significant *F*(2,58) = 1.82, *p* = .172, *η*^2^ = .059.

The interaction between task and orienting modality was significant, *F*(1,29) = 29.2, *p* < .001, *η*^2^ = .502. The tap-contingent gaze latencies in the explicit task (*M* = 296 ms, SD = 26.1) were significantly faster than in the implicit condition (*M* = 333 ms, SD = 37.4), *p* < .001. In contrast the gaze-contingent latencies in the explicit task (*M* = 285 ms, SD = 24.9) did not significantly differ from implicit latencies (*M* = 290 ms, SD = 29.3), *p* = .11.

The interaction between orienting modality and musical IOI was significant, *F*(2,58) = 3.37, *p* = .041, *η*^2^ = .104. Both the gaze-contingent, *F*(2,58) = 5.56, *p* = .006, *η*^2^ = .161, and the tap-contingent gaze latencies *F*(2,58) = 9.84, *p* < .001, *η*^2^ = .253, had a significant main effect of IOI. The latencies slowed linearly as the IOI interval slowed (*p* < .001). The interaction is due to the size of the variance (steepness in the slope) between the two modalities, the variance was greater for the tap-contingent responses.

Finally, the interaction between task and musical IOI was significant, *F*(2,58) = 34.8, *p* < .001, *η*^2^ = .545. The latencies during the explicit task varied significantly by musical IOI *F*(2,58) = 51.7, *p* < .001, *η*^2^ = .641, slowing linearly as the IOI slowed (*p* < .001) with clear evidence for explicit entrainment of eye movements to the music. In contrast, the gaze latencies during the implicit trials did not significantly vary across the IOI levels, *F*(2,58) = .400, *p* < .672, *η*^2^ = .014, rather there is strong Bayesian evidence for the null hypothesis (*BF_01_* = 7.43), and further evidence of invariance across the musical IOI levels, i.e., no evidence of passive entrainment of eye movements to background musical beats. Not only was there no presence effect (compared to silent), there was also no tempi related change.

The above analysis focuses entirely on gaze latency, even in the tap-contingent condition where circle transitions are caused by finger taps, not the gaze shifts. To contextualize the eye movement latencies in the tap-contingent condition above, the finger-tap latencies themselves were also analysed. As with the eye movements during the tap-contingent condition, the implicit finger-tap latencies (*M* = 403 ms, SD = 69.4), were notably slower than the gaze-contingent latencies and showed no evidence of being implicitly entrained by the musical IOI, *BF_01_* = 7.29. The slow latencies of the gaze in the implicit tap-contingent condition (Figure 3, right) were a direct consequence of the slow visual transitions produced by the taps themselves. In the explicit condition the finger-tap latencies were much faster and varied significantly across the IOI levels, *F*(2,58) = 67.0, *p* < .001, *η*^2^ = .698, slowing linearly as the IOI level slowed (264 M = 274 ms, SD = 38.0; 293 M = 305, SD = 17.3; 323 M = 331, SD = 12.9), *p* < .001. These results mirror that of the eye movements in the tap-contingent condition and demonstrate that the timing of eye movements responds to the timing demands of visual information produced by motor actions.

In summary, the latency of eye movements did not implicitly vary subject to the presence of music or with changes in IOI irrespective of the orienting modality. When explicitly synchronizing to the music, the eye movements in the tap-contingent condition varied by IOI to a greater degree (closer approximating the IOI) than the gaze-contingent latencies although both conditions significantly varied linearly in response to the musical IOI.

### Mean resultant length

**Figure 4.**
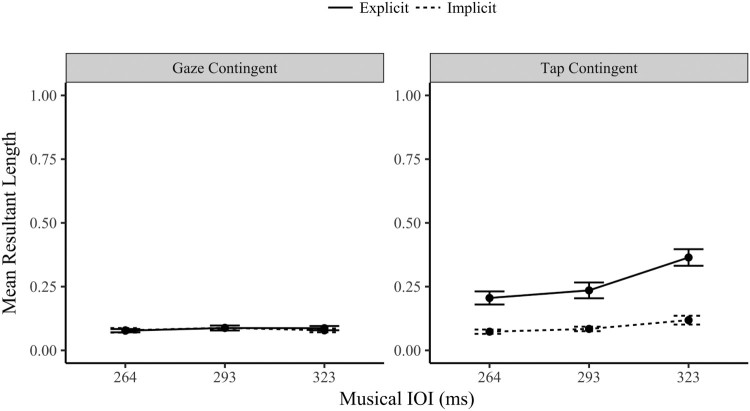
Mean Resultant Length Eye Movement Values (±1 SE) by Orienting Modality (Tap and Gaze Contingent), Task (Implicit and Explicit) and Musical IOI levels (264, 293, 323 ms).

The mean resultant length provides a simple scaled measure of synchronization performance, from 0 (random) to 1 (perfectly synchronized, or very little variation in the angle values). The eye movements were tested for synchronization with the Rayleigh test (Wilkie, 1983). As observed in Table 1, the gaze-contingent conditions had only a small proportion of participants who demonstrated synchronized behaviour. In contrast, the proportion of synchronized tap-contingent gaze responses was much higher. The implicit proportion increased slightly as the musical IOI slowed (to 30% of participants). In the explicit tap-contingent condition a majority synchronized their eye movements (and their finger-taps) during the task. This difference in synchronization ability between the orienting modalities is very apparent in the low MRL values shown in Figure 4, with invariantly poor performance in the gaze-contingent values. It was clearly more difficult to synchronize a gaze-contingent response.

**Table 1.** The proportion of participants with significantly circular eye movement responses by orienting modality, task and musical IOI (significantly non-uniform with the Rayleigh test). The finger-tap values from the tap-contingent condition are also included for reference.

	Gaze synchronization				Tap synchronization	
	Gaze-contingent		Tap-contingent		Tap-contingent	
IOI	Implicit	Explicit	Implicit	Explicit	Implicit	Explicit
264	.10	.03	.03	.63	.17	.83
293	.13	.20	.10	.67	.33	1
323	.03	.13	.30	.90	.43	.97

The following ANOVA should be interpreted with some caution as it is comparing gradations of randomness for some participants (especially in the gaze-contingent conditions). A three-way repeated measures ANOVA compared the gaze MRL values (synchronization) by orienting modality (gaze and tap-contingent), task (implicit and explicit) and musical IOI (264, 293 and 323 ms). The main effects were significant for orienting modality *F*(1,29) =47.0, *p* < .001, *η*^2^ = .618; task, *F*(1, 29) = 39.4, *p* < .001, *η*^2^ = .576; and musical IOI, *F*(2,58) = 15.3, *p* < .001, *η*^2^ = .354. Each of the two-way interactions were also significant (*F* > 14.5, *p* < .001).

The three-way interaction between orienting modality, task and musical IOI was significant, *F*(2,58) = 7.745, *p* < .001, *η*^2^ = .211. To unpack the three-way interaction further, the following analysis separates the data by modality (as shown in Figure 4). A two-way ANOVA of the gaze-contingent MRL values across the three musical IOI and two tasks found no significant main effect of musical IOI, *F*(2,58) = .558, *p* = .575, *η*^2^*^ ^*= .019 (*BF_01_ *= 8.67), or task, *F*(1,29) = .118, *p* = .734, *η*^2^*^ ^*= .004 (*BF_01_ *= 5.96). The interaction between the musical IOI and task was also not significant, *F*(2,58) = .334, *p* = .718, *η*^2^*^ ^*= .011. When considered in combination with the earlier Rayleigh’s test proportions (Table 1), which found poor synchronization in the gaze contingent condition, these MRL results provide strong Bayesian evidence that even though participants altered their gaze latencies in response to musical IOI (as observed in the gaze latency musical IOI effect for the gaze-contingent condition) these changes were not systematic enough to increase the MRL above the random level observed in the implicit condition.

A further two-way ANOVA contrasted the tap-contingent gaze MRL across task and IOI. The main effect of task was significant *F*(1,29) = 46.9, *p* < .001, *η*^2^*^ ^*= .618; the explicit MRL values (*M* = .268, SD = .176) were significantly more synchronized (higher) than the implicit MRL (*M* = .092, SD = .069). The main effect of musical IOI was significant *F*(1,29) = 19.7, *p* < .001, *η*^2^*^ ^*= .405, the MRL values increased linearly as the IOI level slowed (*p* < .001). The interaction between task and musical IOI was also significant, *F*(1,29) = 13.1, *p* < .001, *η*^2^*^ ^*= .311. The MRL values in the explicit task varied significantly by musical IOI, *F*(2,58) = 22.4, *p* < .001*, η*^2^ = .436, improving linearly as the musical IOI slowed (*p* < .001). The implicit MRL values also varied by musical IOI, *F*(2,58) = 4.69, *p* = .013, *η*^2^ = .139, also increasing linearly as the musical IOI slowed (*p *= .005). The interaction is explained by the notable difference in effect size between the two tasks; the steepness of the increase was larger in the explicit task (as well as being a higher MRL overall).

A final analysis of the MRL values is a comparison of the implicit MRL between the orienting modalities. A two-way ANOVA contrasted the implicit MRL values by modality (tap and gaze-contingent eye movements) and musical IOI. The main effect of orienting modality was not significant *F*(1,29) = 1.39, *p* = .247, *η*^2^ = .046 (*BF_01_ *= 3.11). The main effect of musical IOI was marginally significant *F*(2,58) = 2.71, *p* = .075, *η*^2^ = .085 (*BF_01_ *= 1.83). Finally, the interaction between modality and IOI was significant, *F*(2,58) = 3.60, *p* = .034, *η*^2^ = .111. The interaction is explained by the significantly higher MRL in the 323 ms IOI for the tap-contingent eye movements in comparison with the gaze-contingent eye movements (*p *= . 029), i.e., the tap-contingent eye movements show some evidence of implicit synchronization at slower intervals. The remaining two IOI levels were not significantly different between the modalities (*p* > .67). Whilst there was no overall difference in MRL between the implicit conditions, the tap-contingent eye movements at the slowest IOI (323 ms) had a notably higher MRL (and had a higher proportion of participants who synchronized based on the Rayleigh’s test).

### Circular mean

**Figure 5.**
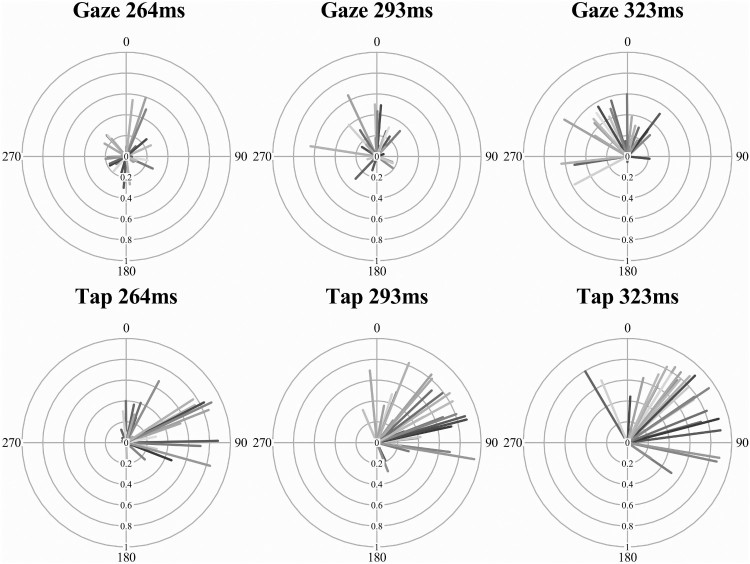
Radial plots of eye movement and finger-tap timing in the explicit tap-contingent conditions across the three IOI levels. The circular mean (line direction) and mean resultant length (line length) is represented for all participants (each a different shade).

An analysis of interest for synchronization behaviour is where the eye movements cluster in relation to the musical beat’s phase, known as the circular mean. This analysis is only meaningful when there is minimal variance in the angles in phase (tested with the Rayleigh’s test). As described above in Table 1, both the explicit tap-contingent eye movements and the finger-taps themselves had a majority of participants with synchronized responses. These two modalities (eye movement and finger taps) from the same explicit tap-contingent condition were analysed and are displayed in the radial plots of Figure 5. The circular mean of each participant (line shading) is represented by the direction of the line (the beat was at 0, i.e.,12 o’clock), early responses (before the beat) are between 180 and 359° and late (after the beat) are between 0 and 180°. The MRL value is the length of the line (from 0 short to 1, a long line).

Fifteen of the participants (half of the sample) had both significantly synchronized eye movements and finger-tap responses in the explicit tap-contingent condition (the Rayleigh’s test was significant for all three IOI). These participants were isolated from the sample for the following analysis of the circular mean. As there is no known within-subject equivalent for circular data, the circular mean was converted to a linear measure of absolute distance (ms) from the IOI (*mean distance*). This was achieved by converting the (radian) circular mean value (which ranged from +π to −π, where 0 was the musical beat) to a degree value, and then re-representing this degree value as a distance (in milliseconds) from the musical IOI.

**Figure 6.**
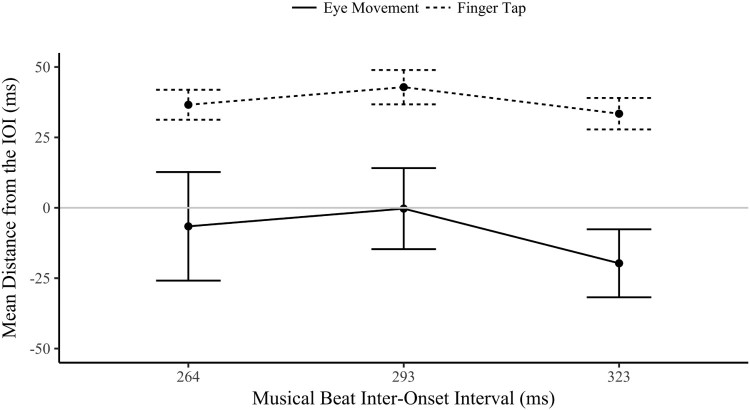
Circular mean distance (ms) ±1 SE from the musical IOI of the eye movements and finger taps in the tap-contingent explicit condition (these finger-taps and eye movements cooccurred in time). A positive value indicates responses after the beat (late), 0 is on-beat and a negative value is before the beat (early).

A two-way ANOVA of the (circular) mean distance was contrasted across the two modalities (eye movement and finger tap) and three musical IOI (264, 293 and 323 ms) for the tap-contingent explicit condition. The main effect of musical IOI was not significant, *F*(2,28) = .785, *p* = .466, *η*^2^ = .053, neither was the interaction between modality and musical IOI, *F*(2,28) = .145, *p* = .866, *η*^2^ = .010. As is clear in the distance between the two lines in Figure 6, the main effect of modality was significant, *F*(1,14) = 44.0, *p* < .001, *η*^2 ^= .759; the finger-taps (*M* = 37.6, SD = 21.8) were significantly later in relation to the musical beat phase than the eye movements (*M* = −8.86, SD = 59.4), which tended to land before the musical beat. These data show that these synchronized eye movements were consistently early in relation to the beat, whilst the tap-responses tended to be later, tapping just after the beat. Although both responses are predictive as reactive movements are classified as being after the onset plus the period required to plan the movement. The most interesting behaviour is the difference between the modalities, both of which were synchronized but independent in phase, with the eyes landing before (anticipating) the finger-tap that produced the visual onset.

## Discussion of experiment 1

The intention of this study was to investigate (1) the entraining influence of music on eye movements, (2) the capacity for intentionally synchronized eye movement and (3) to investigate the nature of eye movements as a rhythmic or predictive behaviour. These three points will be discussed in turn.

### Are eye movements entrained by musical beats and changes in tempi

First, this study found compelling evidence that music did not implicitly influence eye movements, either altering their duration (latency) or promoting phase alignment (MRL), as predicted by the dynamic attending theory (Jones & Boltz, 1989). The timing (and phase) of the music closely aligned with the timing of eye movements during the task (when gaze-contingent), but even with this close relationship, the latencies were robustly invariant in the implicit condition. Secondly, the implicit condition eye movement latencies were much slower during the tap-contingent condition (compared to gaze-contingent), as the visual transitions produced by the finger-taps were also slower than when gaze-contingent. The timing of the eye movements was clearly sensitive to the timing of visual transitions (or the underlying motor action). The latencies in the tap-contingent condition slowed in response to the timing of visual onsets. Therefore, eye movement timing was not generally invariant, rather the music had no influence. In summary, contrary to the predictions of the study that music would implicitly influence and entrain eye movements, we find evidence in favour of the null hypothesis, that eye movement timing is impervious to the presence of music or changes in tempi (supported by Bayes factors). A likely explanation is that intention is required to synchronize, as described in Leow et al. (2018).

### Can eye movements be synchronized with intention?

There are various studies that have tasked horizontal saccades in order to identify the capacity for synchronized eye movements (for example, Hornof & Vessey, 2011; Joiner & Shelhamer, 2006; Shelhamer & Joiner, 2003). A key difference for the present study was that this was an active vision task involving repetitive saccade sequences (the saccade target required visual processing at each location to discriminate letter differences). This more natural eye movement behaviour clearly made it more difficult to synchronize during the gaze-contingent task. However, the gaze-contingent latencies did vary linearly as a response to changes in the IOI during the explicit task. The task, to synchronize with the musical IOI, required only small period corrections, yet the latencies were much slower than the IOI levels, suggesting only gross period correction was achieved. This gross correction was further confirmed by the generally random phase relationship (MRL) of the eye movements to the beat.

An interesting behaviour of note during the experiment was the ability of participants to volitionally quicken their eye movement latencies and move around the circles at a faster speed during the 264 ms explicit gaze-contingent trials. Eye movement timing models, for example, CRISP (Nuthmann et al., 2010), consider direct control as process that can only slow saccade initiation; the engagement of direct control (cognitive demand) inhibits the rate of rise, random walk to threshold, or cancels saccades. The influence of volition or direct control mechanisms as a promoter of eye movements is absent and seemingly contrary to these models. These data suggest it is possible to volitionally quicken the timing of eye movements, without a visual reason to do so (unlike express saccades, e.g., Carpenter, 2001). A plausible explanation is that top-down influence could deemphasise the moment to moment cognitive demands of a task (e.g., by processing less deeply or responding less accurately), which could facilitate a reduction in inhibition and promote faster movements.

Finally, the eye movement latencies during the tap-contingent explicit trials varied significantly by IOI, and were significantly more synchronized than in the gaze-contingent condition. This novel finding is evidence that first, the self-paced gaze-contingent control of eye-movements is less temporally precise than a response to motor-produced visual sequence, and secondly, that eye-movement timing is highly sensitive to and modulated by the timing of rhythmic visual information produced by motor movement.

### Do eye movements predict, react to, or operate independent of visual rhythms?

A novel aspect of this study was the comparison of eye movements between the two orienting modalities. The eye movements in the gaze-contingent condition were not in synchrony with the music and whilst subject to gross shifts in latency (period), they did not show clear evidence of rhythmic timing (phase) even with intent. In contrast, the eye movements in the explicit tap-contingent condition were both synchronized and predictive, either due to the finger-tap produced visual onset (which was mostly synchronized), or the musical beat. A further interesting finding was the mean phase difference of 45 ms between the eye movements (which tended to land early in relation to the musical IOI) and the finger-taps which produced the visual information at the saccade target, which were slightly late. This suggests some predictive accommodation in eye movement timing for future visual events. What is unclear is what is being predicted here, either the music, the visual onset or the motor movement.

## Experiment 2

This second study aimed to further understand the predictive rhythmic saccades observed in the first study’s (E1) tap-contingent trials by clarifying if the timing of these saccades was aligned to the timing of visual onsets (irrespective of source), or the correspondence of music beats, or both? To answer this question the visual search paradigm was modified to produce externally computer-timed visual sequences (of the T and L letters) around the elliptical shape. The timing intervals (*IOI*) were the same as those used as musical IOI in the first study (264, 293, 323 ms) and the presentation *Sequence* was either a constant interval (isochronous, to produce rhythmic eye movements), or random (a random combination of the three IOI intervals). The presence of music (*Audio*) was also manipulated across these conditions. When present it formed audiovisual correspondences with the isochronous sequence to test the additive effect of audiovisual correspondence (Jones & Boltz, 1989; Van der Burg et al., 2008). The music was a competing isochronous auditory rhythm when the sequence was random. A further change was an increase in the number of responses required in the visual search task to incorporate a behaviour measure of reaction time. This change allowed a measure of the perceptual advantage produced by audiovisual onsets, an effect shown by Escoffier et al. (2010). The purpose of these changes was to (1) isolate whether the rhythmic (and predictive) eye movement behaviour also synchronizes to external visual rhythms (as it did for the motor produced tap-contingent onsets), (2) to clarify whether the addition of the music aids this predictive and synchronized behaviour, and (3) to confirm whether audiovisual onsets (letter changes) are identified more quickly than silent or random sequences.

An initial prediction of this second study is that the eye movements in the isochronous conditions will synchronize to the external rhythm as eye movement timing is adaptive and synchronizing rhythmic movements would be beneficial for the task. Secondly, were music aiding the predictive (rhythmic) timing of eye movement behaviour we predict that the isochronous sequence with corresponding music (isochronous music) would show more synchronized eye movements (higher MRL) than when the sequence is isochronous and silent (isochronous silent). Conversely, there would be no difference between the isochronous conditions if only the visual information but not the corresponding music informs eye movement timing.

Finally, if visual attention is entrained by audiovisual onsets, as predicted by the dynamic attending theory (Jones & Boltz, 1989) and shown in Escoffier et al. (2010), the reaction times to letter changes would be fastest with audiovisual correspondence (isochronous music) and slowest with competing musical and visual rhythms (isochronous silent).

### Method

#### Participants

Power analysis using G*Power (Faul et al., 2007) of the rhythmic effect on reaction times identified in Escoffier et al. (2010) utilized their effect size (η^2^* *= .198) to identify a sample size for this study (initial indications suggested *N* = 19, powered to *d *= .95 with *α* = .05). As the eye movement effects can be small, we pre-set the sample size to a compromised value of 24 sufficient to power a medium effect size within the design. The study tested 34 naïve participants and of these 10 were either unable to tap in time (2/10), had some lost eye tracking data in at least one condition (4/10), or had drop in response accuracy below 60% during the experiment (4/10). This left 24 participants (17 Female), with an age range from 20 to 43 (*M* = 24.67, SD = 5.52), who contributed data for analysis. The Gold-MSI (Müllensiefen et al., 2014) musical training scores (on a scale from 7: no training to 49: more training than 95% of the population) indicated a range of training from 8 to 37 (*M* = 22.6, SD = 9.28); none of the participants were professional musicians.  

#### Design modifications

A modification from Experiment 1 (E1) was how the response to changes in visual sequences were collected. Rather than a keyboard key-press that corresponded to the correct letter on screen, the new experiment required simple click of the right trigger on a Microsoft game-controller. This reduced the difficulty of the task by reducing the decision at a letter change. A complete replication of the gaze-contingent condition with this response change was conducted (*N* = 24) and produced the same pattern of results (see the supplementary materials for a summary). As previously described a second modification was how the visual transitions were timed. Rather than being gaze or tap contingent, the timing of transitions was pre-set for the trial. The three musical IOI levels (264, 293 and 323 ms) all remained the same as E1, as did the musical and visual stimuli, screen type, eye-tracker, eye-tracking setup, and data cleaning steps.

#### Procedure

This experiment manipulated the timing of the letter’s visual presentation around the ellipse and the presence of a corresponding (competing or absent) musical beat. The instructions were to follow the visual sequence of letters closely as they move around the circles and click the right trigger on a game controller quickly when the sequence of letters changed (between T and L). The changes in letter sequence occurred much more frequently than in E1, (after 7–13 circles) to increase the number of reaction times collected in a trial and to promote close following of the letter sequence.

The timing of the visual sequence (*sequence*) condition was either isochronous (one of the three IOI levels) or random (the time between each letter transition was randomly one of the three IOI levels). There were two *audio* conditions: First, music that either matched the visual sequence, forming audiovisual correspondences (isochronous music), or added a competing musical IOI when the visual sequence was random (random music). Secondly, a silent condition which formed isochronous silent (the visual sequence was each of the IOIs, there was no music) and random silent conditions. These four conditions were contrasted across the three IOI levels.

**Figure 7.**
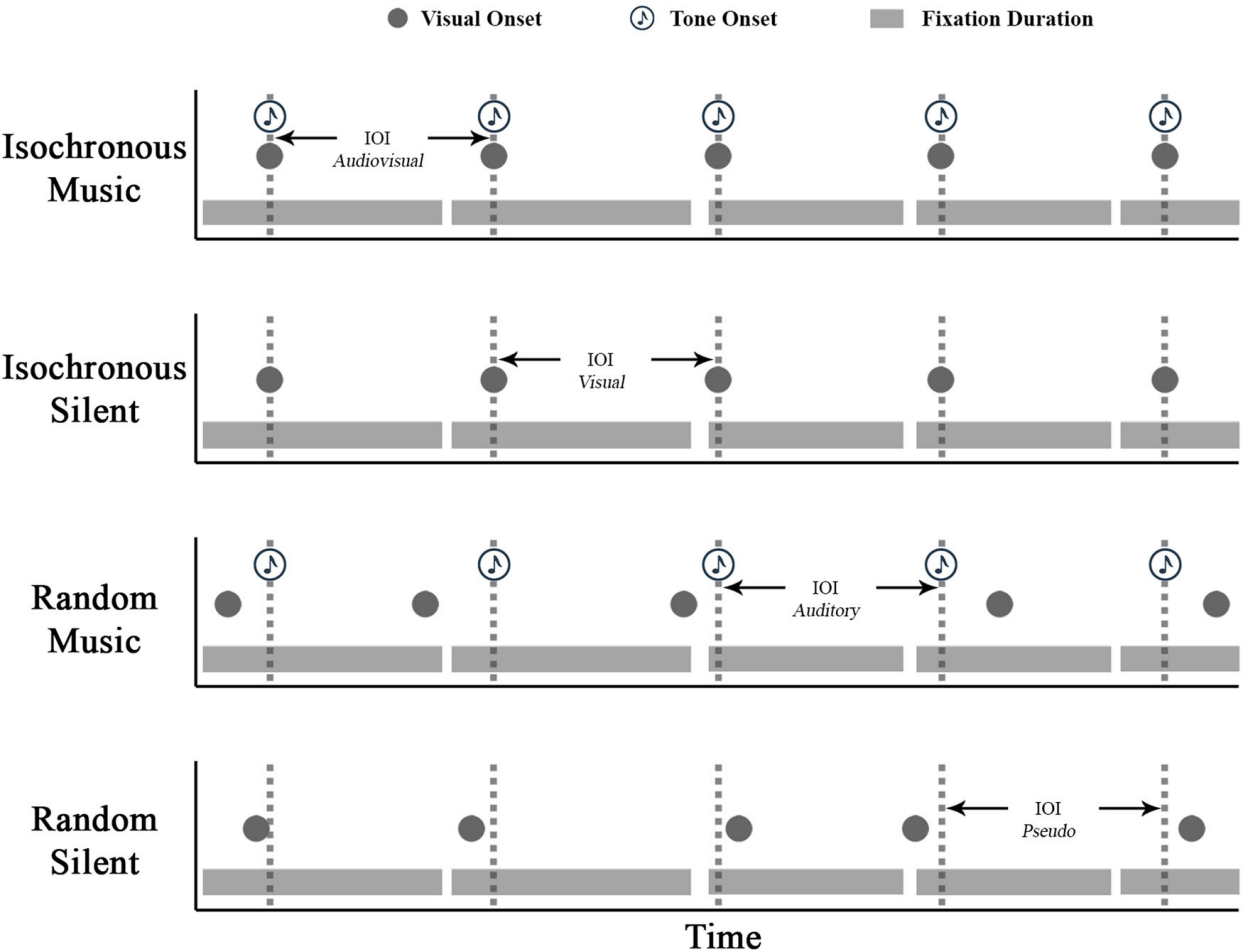
A simulated example of fixation behaviour over time in relation to the IOI (dashed grey line) as an audiovisual, visual, auditory or pseudo-allocated constant through time across the four conditions.

The *IOI* factor varied by which modality presented the isochronous interval (either visual, auditory, i.e., music, or both in the case of isochronous music), but was always one of the three IOI levels as an isochronous referent (264, 293 and 323 ms). When the sequence was random the IOI was the musical beat or a randomized pre-assigned pseudo IOI level when silent (to balance the design), these different IOI representations (especially the random silent pseudo allocation) are shown with simulated fixation behaviour in Figure 7. The mean resultant length value was calculated from the radian difference between the fixation start time and the IOI. For the pseudo-allocated IOI in the random silent condition, the IOI period was a constant (at one of the three intervals) and had no relation to the stimuli presentation.

Each of the twelve trial types, sequence (2 levels) × audio (2 levels) × IOI (3 levels), were repeated three times. The trials were randomized within each block of twelve. Breaks were provided after twelve trials or if the participant moved from the chinrest. Each trial ended after 10 letter sequence changes (on the 11th change to allow a reaction time to all 10 changes to be collected). Following each trial (including the practice) participants were presented their accuracy in detecting the letter changes (accurate detections minus false alarms, as a percentage of all responses). If this detection accuracy was above 60%, the experiment continued with the written encouragement to aim for 100% on every trial. If accuracy dropped below 60% the experiment was held for the experimenter, who terminated it and paid the participant for their time. The task was simple and off-task behaviour (evidenced by low accuracy), indicated a loose following of the visual transitions, which impacted on the quantity of usable data.

The experiments ended with six simple tapping trials on an Apple Magic Trackpad^©^ with each IOI repeated randomly twice. As per the previous experiment each trial collected 60 taps. The final task was to complete the musical training questionnaire and demographic questions.

### Results

#### Reaction time

**Figure 8.**
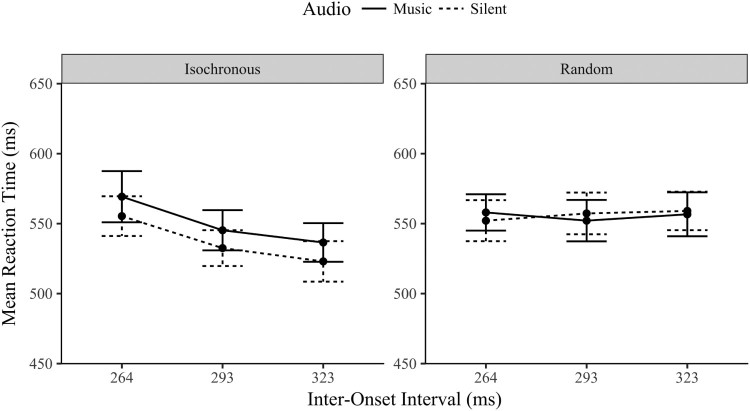
Mean reaction time (ms ± 1SE) by Sequence, Audio and IOI conditions.

A three-way repeated measures ANOVA compared the reaction times (RT) across sequence, audio and IOI conditions. The main effect of sequence was significant, *F*(1,23) = 12.0, *p* = .002, η^2^* *= .343, the isochronous reaction times were quicker (*M* = 544 ms, SD = 72.6) than random (*M* = 556 ms, SD = 69.8) which confirms that the predictable visual onsets were followed more reliably than random ones. The main effect of audio was not significant, *F*(1,23) = 2.47, *p* = .129, η^2^* *= .097. The main effect of IOI was significant, *F*(2,46) = 6.148, *p* = .004, η^2^ = .211, but this is likely due to the marginal significant sequence by audio interaction, *F*(1,23)* *= 3.64*, p *= .069, η^2^* *= .137. displayed in Figure 8.

Analysis of the effect of audio between the two sequence types confirmed the difference observed between the two plots in Figure 8. The RT in the isochronous sequence were quicker when silent (*M* = 537 ms, SD = 63.7) than with music (*M* = 550 ms, SD = 72.1), *t*(23) = 2.12, *p *< .045, *d* = .433. This is in the opposite direction predicted as the synchronized audiovisual presentation was predicted to quicken RT performance. The random sequence did not differ between the audio conditions, *t*(23) = .123, *p *= .903, *d* = .025 (*BF_01 _*= 4.63), which again is contrary to the prediction, that competing audiovisual rhythms would impair (slow) RT performance.

The interaction between sequence and IOI was significant, *F*(2,46) = 7.81, *p* = .001, η^2^* *= .254. The reaction times to isochronous sequences varied significantly by IOI, *F*(2,46) = 14.9, *p *< .001, η^2^* *= .394, RT’s quickening linearly as the IOI slowed (*p* < .01). The RT for the randomly timed visual sequences did not vary across the IOI levels, *BF_01 _*= 7.96 (*F* < 1, *p* > .5), which is unsurprising considering these are pseudo-IOIs and each level was actually made up of a random selection of all three IOI levels. Finally, neither the interaction between the audio and IOI conditions or the three-way interaction between sequence, audio, and IOI were significant (*F* < .3, *p* > .7).

#### Gaze latency

A three-way repeated measures ANOVA compared the latency of eye movements across the sequence (isochronous and random), audio (music and silent) and IOI (264, 293, 323 ms) conditions. There was no main effect of sequence, *F*(1,23) = .259, *p* = .616, η^2^ = .011, a significant main effect of audio, *F*(1,23) = 17.6, *p* < .001*,* η^2^* *= .434; and a significant main effect of IOI, *F*(2,46) = 176, *p* < .001, η^2^* *= .885. The three-way interaction between sequence, audio and IOI was not significant (*F* < .5, *p* > .6).

**Figure 9.**
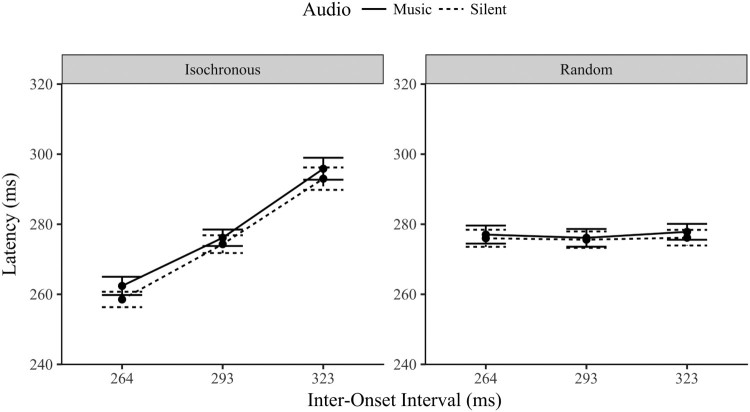
Mean latency of eye movements (±1SE) by Sequence, Audio and IOI.

The interaction between sequence and audio was significant, *F*(1,23) = 5.39, *p* = .029, η^2^* *= .190. Analysis of the effect of audio between the two sequence types confirmed the trend in Figure 9, that the isochronous sequence had slower eye movement latencies with corresponding musical beats (*M* = 278 ms, SD = 12.0) than when silent (*M* = 275 ms, SD = 11.8), *t*(23) = 4.95, *p *< .001, *d *= 1.03. The random sequence did not significantly differ between the two audio conditions, *t*(23) = 1.73, *p *= .097, *d* = .361. Note that only the audiovisual correspondences were slowed (3 ms), the effect of music was not simply a presence of music effect as it did not significantly influence the eye movements during the random sequence.

The interaction between sequence type and IOI was also significant, *F*(2,46) = 124, *p* < .001, η^2^* *= .843. The isochronous sequence varied significantly by IOI, *F*(2,46) = 180, *p* < .001, η^2^* *= .887, as eye movement latencies slowed linearly as the IOI slowed (*p *< .001). The random sequence had positive evidence for invariance across the IOI levels, *BF_01_*_ _= 4.36 (F < 1, *p* > .4), which is unsurprising considering the use of pseudo IOI allocation in the random silent condition meaning the moment-to-moment prediction of the timing onset was near impossible.

#### Mean resultant length

The Rayleigh test was performed for each level of Sequence, Audio and IOI conditions. As observed in Table 2, the majority of participants showed significantly synchronized eye movements when the visual sequence was isochronous (all participants when the IOI was 323 ms). Compared to E1, the use of a perfectly isochronous sequence (as opposed to a tap-generated one) has further improved the synchronization of eye movements.^2^

**Table 2.** The proportion of participants with significantly circular data by modality, task and IOI (significantly non-uniform with the Rayleigh test).

	Isochronous		Random	
	Music	Silent	Music	Silent
264 IOI	.83	.83	.21	.21
293 IOI	.88	.92	.38	.42
323 IOI	1	1	.17	.08

A three-way repeated measures ANOVA contrasted MRL across sequence, audio and IOI conditions. There was a significant main effect of sequence, *F*(1,23) = 97.7, *p* < .001, η^2^* *= .809, no significant main effect of audio, *F*(1,23) = .564, *p* = .460, η^2^* *= .024*,* and a significant main effect of IOI *F*(2,46) = 10.5, *p* < .001, η^2^* *= .314.

**Figure 10.**
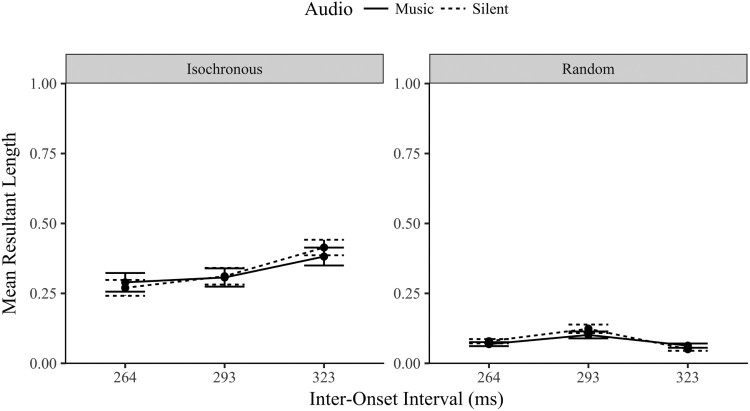
Mean Resultant Length (±1 SE) by Sequence, Audio and IOI conditions.

As shown in Figure 10, the three-way interaction between sequence, audio and IOI was significant, *F*(2,46) = 3.376, *p* = .043, η^2^* *= .128. For the isochronous sequence, there was no main effect of audio, *F*(1,23) = .166, *p* = .687, η^2^* *= .007; the addition of music did not increase the synchronization of eye movements as predicted (*BF_01_* = 5.259). The MRL values did significantly increase linearly as the IOI slowed *F*(2, 46) = 22.5, *p* < .001, η^2^* *= .495, unsurprising considering the fast-paced tempi. There was no significant interaction between audio and IOI (*F* < 2, *p* > .15).

For the random sequence, there was again no significant main effect of audio, *F*(1,23) = 1.03, *p* = .320, η^2^* *= .043 (*BF_01_* = 4.28). The interaction between IOI and audio and the main effect of IOI within the random sequence is meaningless. The IOI was either the musical beat with random music (note that music had no effect) or a pseudo-allocated interval in the random silent condition (not a meaningful measure).

#### Circular mean

**Figure 11.**
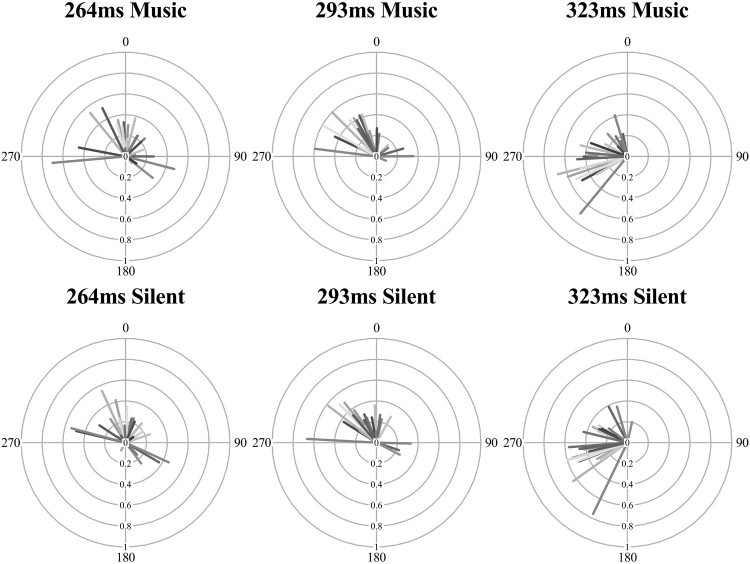
Mean direction (phase location, 0 is the visual or audiovisual onset) and mean resultant length (line length where 1 is the circle radius) by audio condition and IOI (shaded lines represent participants) for the isochronous sequence (values to the right of 0, e.g., 1–90 indicate fixations beginning late, values to the left of 0, e.g., 270–359, indicate early fixations).

As the isochronous trials had significantly synchronized mean resultant length values (Rayleigh’s test), further analysis of the mean phase direction is informative for understanding when in relation to the visual phase the eye movements landed. There is no known within-subject ANOVA equivalent for circular data, shown as radial plots in Figure 11, the circular mean (the phase location of the line) was again converted to a linear measure of absolute distance from the IOI, the mean distance (as described in E1). Note that predictive timing is classified as anticipatory movement, which also incorporates an onset if it begins prior to the period required to plan or produce a movement after the onset (the visual onset time plus the planning period).

**Figure 12.**
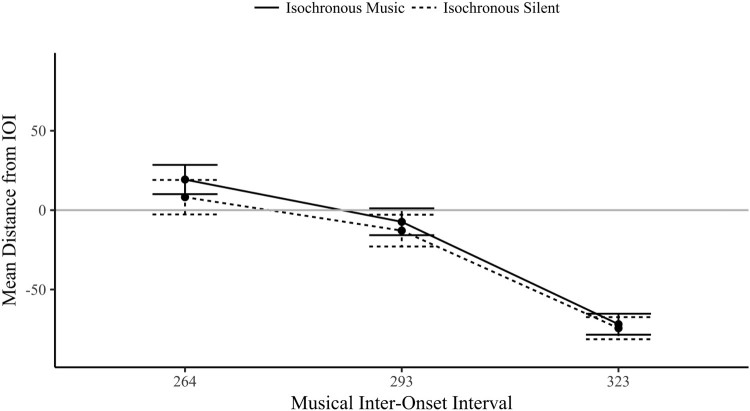
The circular mean distance (ms) of the isochronous conditions by IOI (the IOI is either audiovisual in isochronous music, or visual in isochronous silent).

A two-way repeated measures ANOVA compared the mean distance across audio and IOI isochronous conditions, visualized in Figure 12. There was no significant main effect of audio, *F*(1,23) = 2.14, *p* = .157, η^2^ = .085 (*BF_01_* = 4.17); the presence of musical beats did not influence where in time fixations started in relation to the IOI phase (see the closeness of the two conditions in Figure 12). There was a significant main effect of IOI, *F*(2,46) = 183, *p* < .001, η^2^ = .888, as fixations were increasingly early in response to the visual onset as the IOI levels slowed. The 264 ms IOI (*M* = 13.7 ms, SD = 49.1) had slightly late (although still predictive) response (after the letter onset); the 293 ms IOI (*M* = −10.1 ms, SD = 45.0) and 323 ms IOI (*M* = −73.1 ms, SD = 32.9) had increasingly early responses in phase (Bonferroni corrected pairwise comparisons between the conditions were all significant, *p* < .001). The interaction between audio and IOI was not significant, *F*(2,46) = .352, *p* = .705, η^2^ = .015. The predictive behaviour in eye movements was not aided by the addition of corresponding musical beats, but was a rhythmic response to the temporal demands of predictable visual onsets.

## General discussion

The current understanding of eye movement timing is modelled from viewing behaviour to static scenes (e.g., Engbert et al., 2005; Nuthmann et al., 2010; Tatler et al., 2017). A notable omission of this approach is that the world is dynamic, objects, including the viewer, move. When the eyes move can be the product of volitional action, e.g., as a social signal or intentionally orienting to a peripheral location; but it can also be a response to events produced by motor actions (e.g., when operating machinery), or responding to movement in the world (e.g., when watching sport). The timing of eye movements under these conditions represent distinctions within visual behaviour that only emerge when considering dynamic scenes. An important motivation for this research is that dynamic scenes are inherently multimodal. When the eyes move could be influenced by auditory and or audiovisual information.

The intention for these studies was to investigate eye movements under different visual orienting conditions to measure, (1) whether predictable auditory information, specifically musical beats, are an entraining influence on eye movements; (2) to measure the capacity of intention to produce synchronized gaze-contingent eye movement; and (3) to investigate the rhythmic nature of the saccadic timer as a phased and potentially predictive response to controlled dynamic visual and audiovisual sequences. These points will be discussed in turn, combining the results from both experiments.

### Does music implicitly entrain eye movements and can they be synchronized with intention?

The musical IOI levels employed in this study were chosen to match the average eye movement latencies used to complete the gaze-contingent task in silence in order to minimize the changes in latency (period) and phase required to align with the beat. Employing closely related beat intervals has previously implicitly entrained motor (gait) movements (Moens et al., 2014). The Bayesian evidence from the latency and MRL data in the implicit conditions of experiment 1 found positive evidence for the null hypothesis. Not only did the eye movements not vary between the IOI levels, during these implicit trials, there was no presence of music effect (comparing the latencies to the silent trials). As described in the discussion for experiment 1, in the paradigm used here the eye movements appear impervious to musical influence, either the presence of background music or changes in its tempi.

A recent paper by Leow et al. (2018), argues that intention to synchronize motor movements is required to reliably see changes in behaviour. The present study found that when explicitly synchronizing eye movements during the gaze-contingent task, only gross shifts in period (latency) were achieved with no reliable phase alignment (MRL). Even with intention, the temporal control of eye movements at these fast intervals was poor. As the key mechanisms for direct control are either the cancellation or inhibition of saccades (Nuthmann et al., 2010), it is plausible that slower intervals that allow more time for these (delaying) mechanisms could produce more synchronized behaviour. These direct control limitations could also be a product of the perceptual smoothing of time in visual perception. The visual system presents a temporally smooth visual percept, both of visual events in the world (Yarrow, Haggard, Heal, Brown, & Rothwell, 2001; Yarrow, Haggard, & Rothwell, 2010) and by reducing awareness of saccades that would interrupt the smoothed perceptual stream of visual information (Clarke et al., 2016). This perceptual smoothing appears to limit the visual feedback on when fixations begin, which during a gaze-contingent task could impair the precision of corrective movements. A further limitation for direct control is the tendency to misreport making a directional saccade up to 250 ms prior to it occurring (Deubel, Irwin, & Schneider, 1999). This perceived offset between intention and saccade (action) may also contribute to the observed imprecision in explicitly controlled saccades.

### Are eye movements rhythmic and predictive of visual and audiovisual onsets?

Recent models of eye movement timing, for example, SWIFT, CRISP, or LATEST (Engbert et al., 2005; Nuthmann et al., 2010; Tatler et al., 2017), have produced models of eye movement timing that account for viewing behaviour when reading or viewing static scenes. Each of these models account for variance in fixation duration through dynamic mechanisms that react to the current demands at fixation (e.g., visual complexity or competition in target selection for the next saccade). When objects in the world do not move, these reactive mechanisms (e.g., inhibition, cancellation) can effectively account for behaviour. By omitting scene dynamics, these models have not accounted for the rhythmic and predictive capacity of eye movements, i.e., how vision is able to orient to the right place at the right time. In both the tap-contingent trials of experiment 1 and the externally timed trials in experiment 2, we have shown evidence that the timing of eye movements is highly sensitive to the timing of visual onsets. Eye movements even at these fast intervals can be rhythmic, synchronized and predictive (landing prior to the onset) of visual events. Similar predictive eye movement behaviour has been identified during scene viewing (Henderson, 2017) and in real-world visuomotor tasks, for example when playing sport (Diaz, Cooper, Rothkopf, & Hayhoe, 2013; Hayhoe, McKinney, Chajka, & Pelz, 2012).

In the first experiment we found that eye movements in the tap-contingent condition varied in response to the timing of the tap-produced visual onsets. For example, in the implicit trials the tap latencies and resulting eye movements were much slower than the gaze-contingent latencies. The visual information at fixation between the gaze and tap-contingent conditions was the same. The delay in latency was a modulation in saccade timing for the slower visual onsets. When the explicit tap-contingent trials produced quicker visual onsets in response to the musical IOI, the eye movements not only varied in latency, but also produced rhythmic and synchronized movements that predicted either the tap, the music or the visual onset. In the second experiment, we clarified this ambiguity with externally timed visual sequences, and showed that the synchronization and predictive behaviour in eye movements is in response to visual onsets, and is not informed by corresponding musical beats.

A notable finding was the small but constant (3 ms) slowing in eye movements during the isochronous music condition compared to isochronous silent of experiment 2. This increase in duration is suggestive of difference in eye movement timing that is produced by audiovisual temporal correspondences. This effect was not present with the addition of music to the random sequence or in the gaze-contingent data with and without music. This effect may be evidence that exogenous audiovisual temporal correspondences require more processing time during fixation, a delay incurred when binding an audiovisual representation. A similar effect occurred with the reaction times. When the visual sequence was predictable (isochronous), the reaction times were faster, i.e., the visual sequence entrained visual attention. Interestingly, in addition to slowing eye movements, the corresponding musical beats slowed reaction times uniformly across the IOI levels. The correspondence of audiovisual onsets, rather than entraining the allocation of attention in time (Escoffier et al., 2010; Jones & Boltz, 1989), appear to have increased the cognitive demands of visual processing at the visually entrained moments in time.

### Limitations and future directions

A key strength of the continuous visual search paradigm used here is that it encouraged long sequences of highly systematic saccade programmes, allowing any subtle influences of audio or visual rhythms to be identified. Such saccade sequences more similarly match the patterns of eye movements observed during naturalistic scene viewing (Smith & Henderson, 2009; Tatler & Vincent, 2008) than the short sequences often used (e.g., Hornof & Vessey, 2011; Joiner & Shelhamer, 2006; Shelhamer & Joiner, 2003). However, the use of this highly constrained paradigm may also limit the generalizability of our findings to other eye movement tasks. Future studies should endeavour to replicate these findings (including our support for the null effects e.g., implicit audio influences) in other naturalistic paradigms whilst still controlling known factors influencing saccade dynamics, e.g., task and visual processing demands.

A factor not analysed in this study was the timing of eye-blinks. There is both evidence of interpersonal synchrony of blinks between participants when viewing videos (Nakano, Yamamoto, Kitajo, Takahashi, & Kitazawa, 2009), as well as recent work that describes variance in visual processing aligned with blink onsets (Ang & Maus, 2018). Whilst outside the scope of this paper, which specifically timed the music to align with saccadic intervals, the timing of eye-blinks could be a viable candidate for entrainment.

Although, the eye movements to the visual sequences that were externally timed or tap-generated were generally synchronized and predictive of the visual interval the level of synchronization (the MRL value) was much lower than that produced by motor movements (e.g., a MRL of .73 in Konvalinka, Vuust, Roepstorff, & Frith, 2010). Not all eye movements were synchronized. These data are further evidence in support of mixed control of eye movements, i.e., some eye movements responded to the visual demands of a task and some do not (e.g., Henderson & Smith, 2009; Luke, Nuthmann, & Henderson, 2013; Morrison, 1984). Considering the previously discussed static scene limitation of the timing models, future work could look to identify and quantifying these two distributions in light of visual dynamics, e.g., the source of randomization (a stochastic timer as described by Nuthmann et al., 2010 or a “maverick” in the LATEST model by Tatler et al., 2017) and the predictive and responsive fixations movements that are representative of visual demands.

### Conclusions

The findings of these studies provide evidence that eye movement timing is not only sensitive to and predictive of motor produced and exogenous visual onsets, but is systematically slowed by audiovisual correspondences. We also provide evidence that eye movements can both vary in timing and synchronize with a regular interval when the visual processing demands of the task require it but not to purely auditory rhythms. Finally, we confirm that the direct control of eye movements is limited and much less precise than eye movements in response to motor-produced and exogenous visual onsets. None of these effects are currently accounted for within eye movement timing models.

## Notes

1. The outer locations did not produce a significant delay due to direction change.

2. The relatively high proportion of significant participants in the random 293 ms condition is likely a product of the random timing having a mean interval of 293 ms (each of the three IOI values were randomized to form the random sequence). The larger than expected number of synchronized participants in the random silent condition may be evidence that eye movement timing varies as a product of the average visual timing demands (producing a phase relationship with the pseudo allocated 293 ms interval).
